# Validation of surgical care quality indicators in the Brazilian Unified Health System

**DOI:** 10.11606/s1518-8787.2023057004723

**Published:** 2023-04-12

**Authors:** Anna Cláudia Sales Gomes Caldas, Rafael Santiago de Araújo, Paulo José Medeiros, Marise Reis de Freitas, Victoriano Soria Aledo, Zenewton André da Silva Gama

**Affiliations:** I Secretaria de Estado de Saúde Pública do Rio Grande do Norte Natal RN Brasil Secretaria de Estado de Saúde Pública do Rio Grande do Norte. Natal, RN, Brasil; II Universidade Federal do Rio Grande do Norte Natal RN Brasil Universidade Federal do Rio Grande do Norte. Natal, RN, Brasil; III Universidade Federal do Rio Grande do Norte Departamento de Medicina Integrada Natal RN Brasil Universidade Federal do Rio Grande do Norte. Departamento de Medicina Integrada. Natal, RN, Brasil; IV Universidade Federal do Rio Grande do Norte Departamento de Infectologia Natal RN Brasil Universidade Federal do Rio Grande do Norte. Departamento de Infectologia. Natal, RN, Brasil; V Universidad de Murcia. Facultad de Medicina Murcia España Universidad de Murcia. Facultad de Medicina. Murcia, España; VI Universidade Federal do Rio Grande do Norte Departamento de Saúde Coletiva Natal RN Brasil Universidade Federal do Rio Grande do Norte. Departamento de Saúde Coletiva. Natal, RN, Brasil

**Keywords:** Quality of Health Care, Quality Indicators in Health Care, Patient Safety, Surgical Operating Procedures

## Abstract

**OBJECTIVE:**

To validate a set of indicators for monitoring the quality of surgical procedures in the Brazilian Unified Health System (SUS).

**METHODS:**

Validation study developed in 5 stages: 1) literature review; 2) prioritization of indicators; 3) content validation of indicators by RAND/UCLA consensus method; 4) pilot study for reliability analysis; and 5) development of instruction for tabulation of outcome indicators for monitoring via official information systems.

**RESULTS:**

From the literature review, 217 indicators of surgical quality were identified. The excluded indicators were: those based on scientific evidence lower than 1A, similar, specific, which corresponded to sentinel events; and those that did not apply to the SUS context. Twenty-six indicators with a high level of scientific evidence were submitted to expert consensus. Twenty-two indicators were validated, of which 14 process indicators and 8 outcome indicators with content validation index ≥80%. Of the validated process indicators, 6 were considered substantially reliable (Kappa coefficient between 0.6 and 0.8; p < 0.05) and 2 had almost perfect reliability (Kappa coefficient > 0.8, p < 0.05), when the inter-rater agreement was analyzed. One could measure and establish tabulation mechanism for TabWin for 7 outcome indicators.

**CONCLUSION:**

The study contributes to the development of a set of potentially effective surgical indicators for monitoring the quality of care and patient safety in SUS hospital services.

## INTRODUCTION

The Brazilian Unified Health System (SUS) performs about five million surgeries annually, mostly elective surgical procedures^1^. Such therapeutic resource has been increasingly regarded as an essential component of public health, its role growing in importance with the increase in life expectancy^2^. However, little is known about the quality and safety of surgeries performed in SUS.

This is a crucial gap since, despite their benefits, surgeries also present risks to the patient and costs to the health system. Data shows 312.9 million surgeries were performed in 2012 worldwide^2^, an increase of about 36.8% since the launch of the Second Global Challenge for Patient Safety, Safe Surgeries Saves Lives^3^.

The Ministry of Health, health sector regulatory agencies and non-governmental bodies have supported initiatives to improve the quality and safety of surgeries through actions related to the elaboration of public policies^4^, technical standards and regulations for inspection and monitoring purposes. However, there is still a lack of a standardized set of indicators for monitoring surgeries in SUS. Such monitoring is important since it enables quality improvement and provides learning to teams, in addition to enabling the development of regulatory capacity, being essential for a good clinical performance^5,6^.

In the last decade, indicators have been developed to guide initiatives for improvement of quality in perioperative care^7,8^and to stimulate positive changes towards achieving quality at a reasonable cost^9^. These indicators are used as direct measures of the quality and safety of the care provided; however, they are still insufficient. Therefore, we are dealing with a scenario in which the existing indicators are not standardized and consolidated, nor periodically measured by the care network, leading to a void of important information and lack of comparability between existing information, negatively affecting the planning and quality management of care in the SUS.

Thus, this study aims to identify and validate a minimum set of process and outcome indicators that can be used to monitor the quality of surgical procedures in SUS.

## METHODS

This study is part of the QualiCir Project, an intervention project aimed at improving the quality and safety of surgical procedures in the state of Rio Grande do Norte (RN), and is developed in partnership with the QualiSaúde Research Group of the Federal University of Rio Grande do Norte and the RN Public Health Secretariat.

This is a methodological study on the validation of perioperative quality indicators applicable to elective surgical procedures performed in SUS. The study was developed in 5 stages: 1) literature review; 2) selection of indicators for consensus; 3) content validation of indicators; 4) pilot study for reliability analysis; and 5) development of instructions for tabulation of outcome indicators.

**Stage 1 - Literature review:** A search was performed in *PubMed* and Google Scholar databases, looking for articles of current systematic reviews (< 5 years of publication). As search strategy, the keywords “quality indicators” and “surgical procedures” were included. Searches were also carried out on official State websites and documents, pursuing indicators developed by national organizations regarded as reference in the promotion of patient care and safety, so to obtain a list of potential indicators to be used to measure surgical quality in the Brazilian context. Indicators were selected from regulatory agencies in the health sector^10,11^, Patient Safety Indicators (ISEP-Brazil Project)^12^, Health System Performance Assessment Project (PROADESS)^13^, and the Collaborating Center for Quality and Patient Safety (PROQUALIS)^14^.

**Stage 2 - Selection of indicators for consensus:** Based on the indicators found in the previous step, those that had the following criteria were selected: a) aspects related to the entire surgical process; b) high scientific evidence (1A); c) able to evaluate the quality of surgical care in any hospital of the national health system; d) can be used to implement improvement measures based on their results. Indicators that were similar amongst themselves, sentinels, not applied to the SUS context, that evaluate a specific surgical procedure or patient group, with contradictory evidence, and indicators that present measurement difficulties (many components of measurements, unclear) were excluded.

**Stage 3 - Content validation of the indicators:** Validation was performed using the RAND/UCLA method^15^, which associates aspects of the Delphi and Nominal Group methods^14^ and combines the observation of the available scientific evidence with the collective judgment of experts. The validation of indicators is done through a consensus opinion derived from a group, with aggregated individual opinions, which is an established approach for the development of health indicators^5^. The group of specialists consisted of eight surgeons and two nurses. Nine members of this group of specialists worked in public institutions in four different Brazilian states, and one was a Spanish surgeon who coordinated a similar study in his country.

Two rounds of consensus were established: the first occurred by completing the electronic questionnaire sent by email and the second was developed by web conferencing.

A questionnaire was developed using the *Google Forms platform,* based on similar studies^12,14,16^, containing five closed questions for each indicator, using a Likert-type scale for responses. The following criteria were used for the evaluation and selection of indicators: 1) Is the indicator clearly relevant?; 2) Does the indicator measure the quality of care or safety in surgical care?; 3) Can the indicator be modified with improvement interventions implemented by the hospital?; 4) Are the data for the indicator measurement possible to collect?; and 5) Is the wording of the indicator clear, with correct terminology and leaving no doubts?

Indicators that obtained a content validation index (CVI) greater than 80%^17^ in the five proposed items would be considered valid for the measurement of surgical quality. Indicators that did not reach this value in the first round were taken to the second round.

As a subsidy for the two rounds, an indicator form was developed containing the following information: title, measure, justification, indicator type, data source, numerator and denominator description, clarifications/definition of terms, limitations/exceptions, and bibliographic references.

**Stage 4 - Pilot study for reliability analysis:** For reliability analysis of process indicators, a pilot study was carried out in a hospital of the RN state health network. Three samples were established from the set of surgeries described in the Management System of the Table of Procedures (SIGTAP) of SUS. **Sample 1 (A1):** All procedures of the surgical procedures group, except the subgroups of minor surgeries and surgeries of the skin, subcutaneous tissue and mucosa, upper airway surgery, vision apparatus surgery, obstetric surgery and other surgeries; **sample 2 (A2):** Surgical procedures of the subgroup digestive tract surgeries (colon and rectum surgeries); **sample 3 (A3):** Surgical procedures of the osteomuscular apparatus subgroup surgeries (arthroscopy and knee prosthesis).

Collection was carried out by two independent evaluators, with previous experience in collecting data from medical records, in a cross-sectional manner, in samples of 30 medical records each, referring to elective surgeries occurred in 2020, selected systematically^18,19^. The adequacy of indicators by sample type was established by consulting experts. Most of the process indicators were evaluated in sample A1, with the exception of the indicators “Timely removal of surgical nasogastric tubes” and “Early removal of bladder catheter”, which were evaluated in sample 2.

For the analysis of interobserver reliability, the Kappa index was calculated to identify the level of agreement according to the parameters established by Landis and Koch^20^: poor agreement (Kappa < 0.00), mild agreement (0.00 ≤ Kappa ≤ 0.20), fair agreement (0.21 ≤ Kappa ≤ 0.40), moderate agreement (0.41 ≤ Kappa ≤ 0.60), substantial agreement (0.61 ≤ Kappa ≤ 0.80) and perfect agreement (0.81 ≤ 1.00).

**Stage 5 - Identification of tabulation mechanism for result indicators so that they can be monitored via official information systems** - The validated result indicators were analyzed for their possibility of monitoring through the use of data from official information systems, from the identification of tabulation mechanism for TabWin/DataSus with the Hospital Information System of SUS (SIH-SUS - *Sistema de Informações Hospitalares do SUS*) database.

The research was carried out under the approval of the Research Ethics Committee of the Federal University of Rio Grande do Norte (CEP-HUOL, CAAE: 39976920.6.0000.5292), following the ethical precepts in research with human beings, according to resolution CNS/MS 466/12.

## RESULTS

217 quality or safety indicators related to surgical procedures, totaling 183 process indicators and 34 outcome indicators were found. The choice to use the content of systematic reviews as the main reference for the literature search was made to avoid the repetition of a recent study with similar objectives.

**Figure 1:**
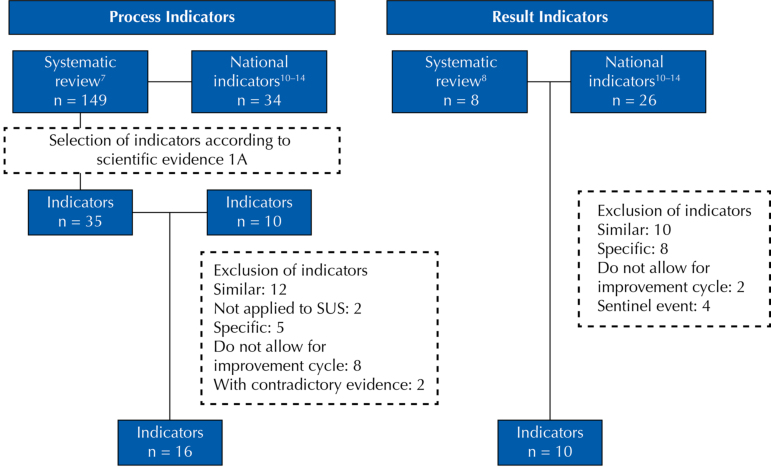
Selection flow of indicators according to exclusion criteria.

Source: Prepared by the authors.

Of the 183 process indicators, 138 were excluded by the criterion of low scientific evidence (< 1A) (Figure 1). Although the level of evidence of the indicator “Use of safe surgery checklist” is not high, the researchers decided to keep this indicator in the study due to its regulation in Brazilian health services. Twelve indicators were excluded because they were considered similar, two because they were not applied to the sus, five because they were indicators applied to a very specific public or procedure, eight did not allow the development of improvement cycles and two were based on contradictory scientific evidence.

As for the outcome indicators, 10 indicators were excluded because they were considered similar, eight were very specific, two did not allow the development of improvement cycles and four were related to sentinel events. At the end of this trial, 16 process indicators and 10 outcome indicators were submitted to content validation with the group of experts. The selection flow of indicators can be seen in Figure 1.

In the first round, which was attended by 100% of the invited experts, validation questionnaires were sent by email and 26 indicators were presented to the group. In this round, the 13 indicators that received CVI greater than 80% were considered valid for measuring surgical quality within the SUS. The other 13 indicators, due to achieving CVI equal to or less than 80% in any of the evaluated criteria, were submitted to the second round of consensus. This step occurred through web conferencing and was attended by 80% of the invited experts. Discussions on indicators with CVI ≤ 80% took place at the time and, subsequently, a new evaluation was carried out, as can be seen in Chart 1.

**Chart 1:** Result of the content validity index obtained in the consensus phases.

Indicator	Content validation index									
	I. Is the indicator clearly relevant?		II. Does the indicator measure the quality of care or safety in surgical care?		III. Can the indicator be modified with improvement interventions implemented by the hospital?		IV. Are the data for the measurement of the indicator collectable?		V. Is the wording of the indicator clear, with correct terminology and leaving no doubts? If not, please suggest change	
Process indicators										
	1st (%)	2nd (%)	1st (%)	2nd (%)	1st (%)	2nd (%)	1st (%)	2nd (%)	1st (%)	2nd (%)
Use of analgesic medication or preoperative sedation through adequate screening	90	100	90	100	100	100	80	87.50	70	87.50
Appropriate use of perioperative morphine	70	87.50	50	87.50	70	100	60	87.50	70	87.50
Screening for postoperative delirium	90	87.50	80	87.50	100	87.50	70	87.50	80	87.50
Control of normothermia in the perioperative period	100		100		100		90		100	
Adequate antibiotic prophylaxis	100		100		100		100		100	
Trichotomy with trimmer or scissors	100	100	90	100	100	100	80	100	100	100
Adequate perioperative venous thromboembolism prophylaxis	100		100		100		100		100	
Early post-surgical ambulation	100	100	100	100	100	100	80	87.50	90	100
Postoperative discharge with postoperative evaluation, prophylaxis of venous thromboembolism and postoperative rehabilitation	100	100	100	100	100	100	80	100	90	100
Preoperative use of oral carbohydrates	80	87.50	90	100	90	87.50	80	75	80	75
Enhanced recovery	90	87.50	90	87.50	90	87.50	70	75	80	75
Release from the oral diet or enteral tube in the first 24 hours	100	100	90	100	90	87.50	90		80	87.50
Timely removal of surgical nasogastric tubes	100		100		100		100		100	
Early Bladder Catheter Removal	100		100		100		100		100	
Pressure and time recording during controlled ischemia in surgery	90		90		90		90		90	
Use of Safe Surgery Checklist	100		100		90		100		100	
Outcome indicators										
	1st (%)	2nd (%)	1st (%)	2nd (%)	1st (%)	2nd (%)	1st (%)	2nd (%)	1st (%)	2nd (%)
Complications related to anesthesia	100	87.50	100	100	80	87.50	90		90	87.50
Perioperative pulmonary embolism or deep vein thrombosis	100		100		90		90		100	
Postoperative sepsis	100		100		90		100		100	
Surgical site infection	100		100		100		100		100	
Post-surgical stroke	80	87.50	80	87.50	70	75	80		90	87.5
Surgical mortality within 30 days	100		100		90		90		90	
Unscheduled admission to intensive care unit	80	87.50	80	87.50	80	75	80		80	87.50
Hospital readmission within 30 days	100	100	100	100	80	87	100		90	100
Length of stay without in-hospital mortality	90		90		90		100		100	
Length of stay with in-hospital mortality	90		90		90		100		100	

Source: Prepared by the authors.

At the end of the second round, four indicators received CVI ≤ 80% and were not considered valid: the indicator “Preoperative use of oral carbohydrates”, which presented CVI of 75% in the criterion related to the writing of the indicator; the indicator “Improved recovery” had CVI of 75% in the criteria related to the availability of data for measurement and clarity in the writing; the indicators “Post-surgical stroke” and “Unscheduled admission to an intensive care unit” obtained CVI of 75% in the criteria related to the availability of data and the possibility of modifying the indicator through improvement interventions. Thus, 22 indicators were considered valid for the measurement of quality in surgeries, of which 14 were process and 8 were outcome indicators. The data source, numerator and denominator of these indicators are described in Chart 2.

**Chart 2:** Indicators validated by experts with description of their respective numerator, denominator and data source.

Indicators	Numerator	Denominator	Data source
1. Use of analgesic medication or preoperative sedation through adequate screening	No. of patients who used opioid analgesics or preoperative sedation to manage preoperative pain and anxiety through adequate screening x 100	No. of patients submitted to the use of anxiolytics or opioid analgesics in the preoperative period	Patient record
2. Proper use of perioperative morphine	No. of patients who used morphine or derivatives by epidural or intraoperative spachymedullary x 100	No. of patients using morphine or intraoperative derivatives	Patient record
4. Control of intraoperative normothermia	No. of adults undergoing surgery with general or regional anesthesia who had normothermia (temperature > 36°) maintained before, during and after surgery x 100	No. of adults undergoing surgery with general or regional anesthesia	Patient record
5. Adequate antibiotic prophylaxis	No. of patients with adequate prophylaxis in all its aspects x 100	No. of surgical patients with indication of surgical antibiotic prophylaxis	Patient record
6. Trichotomy with trimmer or scissors	No. of surgeries that performed trichotomy with trimmer or scissors x 100	No. of surgeries with trichotomy	Patient record
7. Adequate perioperative venous thromboembolism prophylaxis	No. of patients undergoing surgery with indication pharmacological thromboprophylaxis who received appropriate venous thromboembolism prophylaxis initiated within 24h before surgery up to 24h after surgery x 100	No. of surgical patients with indication of pharmacological thromboprophylaxis	Patient record
8. Early post-surgical ambulation	No. of patients who received guidance for early post-surgical ambulation x 100	No. of surgical patients	Patient record
9. Postoperative discharge with postoperative evaluation, prophylaxis of venous thromboembolism and postoperative rehabilitation.	No. of patients who received postoperative evaluation, prophylaxis of venous thromboembolism and postoperative rehabilitation before discharge and who have adequate compliance with the 3 items x 100	No. of patients with postoperative surgery and who were discharged	Patient record
10. Release from the oral diet or enteral tube in the first 24 hours	No. of patients who had the diet released orally or SNE in the first 24h x 100	No. of patients undergoing surgical procedures	Patient record
11. Timely removal of surgical nasogastric tubes	No. of patients who removed the nasogastric tube before the end of surgery	No. of patients submitted to gastrointestinal surgical procedures and who required the use of a nasogastric tube for drainage	Patient record
12. Early Bladder Catheter Removal	No. of patients whose catheter was removed up to 48 hours postoperatively x 100	No. of surgical patients who used a bladder catheter	Patient record
13. Pressure and time recording during controlled ischemia in surgery	No. of interventions with adequate time and pressure recording of pneumatic turnstile X 100	No. of surgical interventions with pneumatic tourniquet	Patient record
14. Use of Safe Surgery Checklist	No. of surgeries with complete performance of the WHO safety checklist x 100	No. of surgeries performed in the institution	Patient record
15. Complications related to anesthesia	Discharges with anesthetic adverse effects, or anesthetic intoxication, recorded in secondary diagnoses, among cases that meet the inclusion and exclusion criteria of the denominator	All surgical discharges, from patients aged 18 years or older	SIH-SUS
16. Perioperative pulmonary embolism or deep vein thrombosis	Discharges, in secondary diagnosis, of deep vein thrombosis or pulmonary embolism among cases that meet the inclusion and exclusion criteria of the denominator x 100	All surgical discharges of patients aged 18 years or older	SIH-SUS
17. Postoperative sepsis	Discharges of sepsis in secondary diagnosis, among cases that meet the inclusion and exclusion criteria of the denominator x 1000	All surgical discharges of patients aged 18 years or older	SIH-SUS
18. Surgical site infection	No. of surgical site infections (within 30 days) x 100	No. of surgeries performed in the period	Patient record
19. Surgical mortality within 30 days	No. of surgical deaths observed in the hospital	No. of surgical procedures performed in the hospital	SIH-SUS
20. Hospital readmission for postoperative complications related to the surgical procedure	No. of patients readmitted between 0 and 29 days of hospital discharge after surgical procedure with complications related to surgery x 100	No. of surgical discharges	SIH-SUS
21. Length of stay without in-hospital mortality	Sum of the number of days each patient discharged without death is hospitalized after a surgical procedure	Sum of the number of patients who were hospitalized after an operative procedure and do not progress to death	SIH-SUS
22. Length of stay with in-hospital mortality	Sum of the number of days each patient spent hospitalized	Sum of the number of patients who were hospitalized after an operative procedure and progress to death	SIH-SUS

SIH-SUS: *Hospital Information System of SUS (Sistema de Informações Hospitalares do SUS*).

The qualification sheets of the validated indicators were reformulated according to suggestions of the experts, with the addition and reformulation of terms and concepts.

To analyze the reliability of the indicators, whose data source are the medical records, a retrospective pilot study was carried out at the Regional Hospital Mariano Coelho (HRMC), in Currais Novos/RN, between September and October 2021. The HRMC has 32 qualified surgical beds, and is a reference in the performance of elective surgical procedures for the health region in which the hospital is inserted.

Due to the HRMC qualification profile, it was not possible to collect the indicators “Postoperative discharge with postoperative evaluation, prophylaxis of venous thromboembolism and postoperative rehabilitation”, and “Record of pressure and time during controlled ischemia in surgery”. The search for another institution of the state hospital network that was qualified to perform orthopedic surgeries to evaluate these indicators was considered; however, this was not possible given the low number of orthopedic elective surgeries performed in 2020 due to the covid-19 pandemic, in addition to the lack of pneumatic tourniquet in the hospital institutions that make up the state network.

As for the reliability analysis, six indicators showed substantial reliability and two almost perfect reliability^20^, as can be seen in Table 1. One could not measure the reliability for the process indicators “Control of normothermia in the perioperative period”, “Screening of postoperative delirium”, “Prophylaxis of adequate perioperative venous thromboembolism” and “Use of safe surgery checklist”, since the percentage of compliance for these indicators was 0% for both evaluators.

**Table 1:** Analysis of the reliability of surgical quality indicators according Landis and Koch (1977) parameters and percentage of compliance achieved.

Variable	Kappaa Index^a^	Classification^b^	Prevalence of the evaluated characteristic
Routine non-administration of anesthetic medication or preoperative sedation	0.73	Substantial	46.7
Appropriate use of perioperative morphine	0.66	Substantial	53.3
Screening for postoperative delirium	-	-	0
Control of normothermia in the perioperative period	-	-	0
Adequate antibiotic prophylaxis	0.62	Substantial	23.3
Trichotomy with trimmer or scissors	1	Almost Perfect	100
Adequate perioperative venous thromboembolism prophylaxis	-	-	0
Early post-surgical ambulation	0.72	Substantial	33
Postoperative discharge with postoperative evaluation, prophylaxis of venous thromboembolism and postoperative rehabilitation	-	-	-
Release from the oral diet or enteral tube in the first 24 hours	0.76	Substantial	80
Appropriate use of postoperative nasogastric tubes (SNG)	0.65	Substantial	96.7
Early Removal of Bladder Catheter	1	Almost Perfect	100
Pressure and time recording during controlled ischemia in surgery	-	-	-
Use of Safe Surgery Checklist	-	-	0
Surgical site infection	-	-	Note: no records of events were observed in the investigated medical records

^a^ For all cases p < 0.001

^b^ Degree of Inter-rater agreement

For outcome indicators, whose data source is SIH-SUS, it was observed that seven of the eight validated indicators can be monitored from the TabWin/DATASUS tabulator. Data are publicly accessible and available at https://datasus.saude.gov.br/transferencia-de-arquivos/.

It was not possible to perform tabulation for the indicator “Post-surgical readmission”. As this is a system that analyzes hospital production, it does not link hospitalizations to an individual user record, i.e., through the system one cannot identify how many times a single user was admitted to the hospital, nor is it possible to ascertain whether one admission would be related to the previous one.

An instruction was prepared to tabulate the result indicators for the TabWin/DATASUS application for teams that will collect data and monitor it. All results obtained with the other indicators can be seen in Table 2.

**Table 2:** Estimates of outcome indicators.

Indicator	Events	Denominator	Outcome
Complications related to anesthesia	0	631 surgical procedures performed	0
Perioperative pulmonary embolism or deep vein thrombosis	0	631 surgical procedures performed	0
Postoperative sepsis	0	631 surgical procedures performed	0
Surgical site infection	0	631 surgical procedures performed	0
Surgical mortality within 30 days	4 post-surgical deaths	631 surgical procedures performed	0.0063
Hospital readmission	-	631	0
Length of stay with in-hospital mortality	48 days with post-surgical deaths	4 discharges with deaths after elective surgical procedure	12 days stay on average
Length of stay without in-hospital mortality	974 days without post-surgical deaths	627 discharges without death after elective surgical procedures	1.5 days stay on average

## DISCUSSION

This study contributed to the development of a set of 22 indicators with a high level of evidence, which underwent a rigorous content validation process to enable the monitoring of the quality of surgical care within the SUS. These indicators can guide the management of institutions and of the hospital network as a whole, identifying weaknesses that must be addressed, aiming at providing safe care to the population. This is, therefore, an initial set of highly relevant indicators for monitoring and improving the quality of surgical care within the scope of SUS RN, with the possibility of being used by any other health service.

From the process indicators, one may evaluate all the steps and activities performed in the implementation of a treatment or care episode^8^. Thus, continuously monitoring these indicators enables one to identify weaknesses in the provision of care. According to Donabedian, process indicators are the only direct measure of quality, as the structure may not be used and outcomes may be due to factors other than good care^21^.

Monitoring of the outcome indicators “Post-surgical mortality”, “Post-surgical readmission” and “Average length of stay with and without death” through the information system enables the measurement of the quality of an isolated health service, as well as *benchmarking*. That is, it enables the comparison of health services from the state hospital network and also at the national level, which strengthens information systems^22^.

The post-surgical mortality indicator is among the indicators proposed by the Lancet Commission^23^ to assess surgical care. A similar study^16^ developed for the Spanish health system also pointed out the indicators: “Post-surgical readmission”, “Prophylaxis of venous thromboembolism”, “Adequate antibiotic prophylaxis” and “Surgical site infection” as valid indicators to assess surgical quality; however, these indicators are directed only to surgeries of the digestive tract.

*Benchmarking* has been used to seek opportunities for improvement and make comparisons of similar organizations^16,24^. It has been listed as a strategy by the World Health Organization (WHO) in the Global Action Plan for Patient Safety 2021-2030^22^, and the development of “good” indicators is a success factor for *benchmarking* actions^25^.

In addition, 11 indicators could be measured with the available data sources (medical records and data from the official information system), of which 8 process indicators were evaluated in medical records and 3 outcome indicators were measured with SIH-SUS data, exploring the feasibility of using this system to evaluate the quality of surgical care. For the indicators “Screening for postoperative delirium”, “Use of safe surgery checklist” and “Prophylaxis of adequate perioperative venous thromboembolism”, one should institutionalize protocols related to these indicators, which signals an opportunity for improvement for the hospital where the pilot was developed.

The inter-rater reliability, tested by Kappa statistics for eight process indicators, found values that characterize a substantial and almost perfect degree of reliability, which reinforces the solidity of these indicators. The Kappa test is considered adequate to evaluate the reliability of inter-rater categorical and nominal variables, and is frequently used to evaluate the reliability in this type of study^20^.

For the Surgical Site Infection (SSI) indicator, whose data sources may be medical records or system data, it was not possible to analyze the reliability, since the event was not observed in the medical records selected to compose the sample. Most SSIs occur, on average, four to six days after the procedure, and the average length of stay for the procedures included in the study was 1.5 days. Studies indicate that, in procedures in which the postoperative length of stay is short, SSI data, obtained only from hospitalized patients, do not reflect the actual occurrence of infection^26^. There was a four-fold increase in SSI when post-discharge surveillance was performed^27^, which leads one to the finding that patient’s medical record does not prove to be the best source of data for monitoring this indicator for the vast majority of procedures performed by the SUS.

For the outcome indicators “Complications related to anesthesia”, “Postoperative sepsis”, “Pulmonary edema or deep vein thrombosis”, measurement via the information system was not possible. The results were null, possibly due to underreporting of secondary events in the Hospital Admission Authorization (AIH) forms. A study on the reliability of AIH data in the country identified a high degree of underreporting of secondary diagnosis^28^. The underreporting of secondary diagnosis in surgical admissions impacts the accuracy of measures calculated for these indicators, which is an opportunity for improvement for the health information system.

The Minimum Health Care Data Set (CMD), conceived in 2015, is a strategy assumed by managers of the three SUS management spheres to reduce fragmentation of information systems, and would replace the main health care information systems in the country. However, despite having been officially instituted by resolution of the Tripartite Intermanagerial Commission^29^, its implementation has not yet been completed. The CMD implementation would enable the use of administrative, clinical-administrative, and clinical data through a single document, in addition to enabling more specific analyzes, since it would relate the information to the identification of users through integration with the base of the National Health Card system. Despite the efforts and studies carried out in the field of patient safety, the ability to reduce risk, avoid harm, and improve health care safety is still hampered by the absence of high-quality information systems^22^.

The review of existing literature and consensus methods are increasingly used and recommended by the scientific community for this type of study^16,30^. The use of the RAND/UCLA method to establish consensus, through the use of remote communication resources (*internet*), allowed to bring together qualified specialists from various regions of the country. The interest of experts in the studied area, associated with the observed consensus indexes, gave credibility to the results, as can be seen in other studies^14,31^.

As limitations of this study, we can highlight the performance of the pilot study in a single hospital, whose care profile did not include surgical procedures of the musculoskeletal system, as well as the conduct of the pilot study in a pandemic period, which decreased the sample universe, due to the cancellation of elective surgeries throughout the hospital network. Other limitations, which may be the subject of further studies, are the non-assessment of structural indicators and the non-performance of the feasibility analysis for the collection of indicators.

## CONCLUSION

This study contributed to the development of a set of quality indicators in the surgical sphere, which translates as an effective mechanism for measuring the performance and quality of care offered by the hospital service network of RN and Brazil. There are 22 indicators that were considered valid, with 8 process indicators considered reliable and seven result indicators, in which parameters were identified for tabulation using the official information systems. This set of indicators enables the documentation of quality of care, enables comparisons and *benchmarking* between health units, promotes the identification of priorities through the strengthening and optimization of monitoring strategies and improvements aimed at patient safety in SUS hospitals.

Therefore, this is an innovative proposal, compatible with the Brazilian reality, to guide public managers and researchers in the process of monitoring surgical quality.
