# Supplementary material for: Validation of surgical care quality indicators in the Brazilian Unified Health System
Source: Rev Saude Publica. 2023 Apr 12;57:27. doi: 10.11606/s1518-8787.2023057004723 (PMC10124162; doi:10.11606/s1518-8787.2023057004723)
Supplement: Translation

# Translation: Validação de indicadores de qualidade do cuidado cirúrgico no Sistema Único de Saúde

**Authors:** Anna Cláudia Sales Gomes Caldas, Rafael Santiago de Araújo, Paulo José Medeiros, Marise Reis de Freitas, Victoriano Soria Aledo, Zenewton André da Silva Gama

**Affiliations:** I Brasil Secretaria de Estado de Saúde Pública do Rio Grande do Norte. Natal, RN, Brasil; II Brasil Universidade Federal do Rio Grande do Norte. Natal, RN, Brasil; III Brasil Universidade Federal do Rio Grande do Norte. Departamento de Medicina Integrada. Natal, RN, Brasil; IV Brasil Universidade Federal do Rio Grande do Norte. Departamento de Infectologia. Natal, RN, Brasil; V España Universidad de Murcia. Facultad de Medicina. Murcia, España; VI Brasil Universidade Federal do Rio Grande do Norte. Departamento de Saúde Coletiva. Natal, RN, Brasil

**Keywords:** Qualidade da Assistência à Saúde, Indicadores de Qualidade em Assistência à Saúde, Segurança do Paciente, Procedimentos Cirúrgicos Operatórios

## Abstract

**OBJETIVO:**

Validar um conjunto de indicadores para monitoramento da qualidade dos procedimentos cirúrgicos no Sistema Único de Saúde (SUS).

**MÉTODOS:**

Estudo de validação desenvolvido em 5 etapas: 1) revisão de literatura; 2) priorização de indicadores; 3) validação de conteúdo dos indicadores por método de consenso RAND/UCLA; 4) estudo piloto para análise da confiabilidade; e 5) desenvolvimento de instrutivo para tabulação dos indicadores de resultado para monitoramento via sistemas de informações oficiais.

**RESULTADOS:**

A partir da revisão de literatura, foram identificados 217 indicadores de qualidade cirúrgica. Os indicadores excluídos foram: indicadores baseados em evidências científicas inferiores a 1A, similares, específicos, que correspondiam a eventos sentinelas; e aqueles que não se aplicavam ao contexto do SUS. Foram submetidos ao consenso de especialistas 26 indicadores com alto nível de evidência científica. Foram validados 22 indicadores, dos quais 14 indicadores de processo e 8 indicadores de resultado com índice de validação de conteúdo ≥80%. Dos indicadores de processo validados, 6 foram considerados confiáveis substancialmente (Coeficiente de Kappa entre 0,6 e 0,8; p < 0,05) e 2 tiveram confiabilidade quase perfeita (coeficiente de Kappa > 0,8, p < 0,05), quando analisada a concordância interavaliador. Foi possível mensurar e estabelecer mecanismo de tabulação para TabWin para 7 indicadores de resultado.

**CONCLUSÃO:**

O estudo contribui com o desenvolvimento de um conjunto de indicadores cirúrgicos potencialmente eficazes para o monitoramento da qualidade do cuidado e segurança do paciente nos serviços hospitalares do SUS.

## INTRODUÇÃO

O Sistema Único de Saúde (SUS) realiza anualmente cerca de cinco milhões de cirurgias, em sua maioria procedimentos cirúrgicos eletivos^1^. Cada vez mais esse recurso terapêutico vem sendo considerado componente essencial da saúde pública e seu papel ganha importância com o aumento da expectativa de vida^2^. Entretanto, pouco se sabe sobre a qualidade e segurança das cirurgias realizadas no SUS.

Essa é uma lacuna importante, pois, apesar dos benefícios, as cirurgias também apresentam riscos ao paciente e custos ao sistema de saúde. Dados mostram que, em 2012, foram realizadas 312,9 milhões de cirurgias no mundo^2^, um aumento de cerca de 36,8% desde o lançamento do Segundo Desafio Global para a Segurança do Paciente, Cirurgias Seguras Salvam Vidas^3^.

O Ministério da Saúde, as agências reguladoras do setor de saúde e os órgãos não governamentais têm apoiado iniciativas para melhorar a qualidade e segurança das cirurgias, através de ações relacionadas à elaboração de políticas públicas^4^, normas técnicas e regulamentações, para fins de fiscalização e de monitoramento. Porém, ainda há uma carência de um conjunto padronizado de indicadores para o monitoramento das cirurgias no SUS. Esse monitoramento é importante, uma vez que possibilita melhoria da qualidade e proporciona aprendizado às equipes, além de possibilitar a melhoria da capacidade regulatória, sendo essencial ao bom desempenho clínico^5,6^.

Na última década foram desenvolvidos indicadores para orientar as iniciativas de melhoria da qualidade para os cuidados perioperatórios^7,8^ e estimular mudanças positivas em relação ao alcance da qualidade a um custo razoável^9^. Esses indicadores são usados como medidas diretas da qualidade e segurança do atendimento prestado; porém, ainda são insuficientes. Entretanto, lidamos com um cenário em que os indicadores existentes não são padronizados e consolidados, ou medidos periodicamente pela rede assistencial, o que nos leva a um vazio de informações importantes e falta de comparabilidade entre as informações existentes, afetando negativamente o planejamento e gestão da qualidade do cuidado no SUS.

Logo, o objetivo deste estudo é identificar e validar um conjunto mínimo de indicadores de processo e resultado que possam ser utilizados para o monitoramento da qualidade dos procedimentos cirúrgicos no SUS.

## MÉTODOS

O estudo faz parte do Projeto QualiCir, projeto de intervenção que visa melhorar a qualidade e segurança nos procedimentos cirúrgicos no estado do Rio Grande do Norte (RN), desenvolvido em parceria com o Grupo de Pesquisa QualiSaúde da Universidade Federal do Rio Grande do Norte e Secretaria de Saúde Pública do RN.

Este é um estudo metodológico de validação de indicadores de qualidade perioperatória aplicáveis aos procedimentos cirúrgicos eletivos realizados no SUS. Foi desenvolvido em 5 etapas: 1) revisão de literatura; 2) seleção dos indicadores para consenso; 3) validação de conteúdo dos indicadores; 4) estudo piloto para análise da confiabilidade; e 5) desenvolvimento de instrutivo para tabulação de indicadores de resultado.

**Etapa 1 - Revisão de literatura:** Foi realizada busca em bases de dados, *PubMed* e Google Acadêmico, por artigos de revisões sistemáticas atuais (< 5 anos de publicação). A estratégia de busca incluiu, como palavras-chave, “indicadores de qualidade” e “procedimentos cirúrgicos”. Também foram realizadas buscas em sites e documentos oficiais nacionais por indicadores desenvolvidos por organizações nacionais consideradas referência na promoção do cuidado e da segurança do paciente, na perspectiva de se obter uma lista de potenciais indicadores que possam ser utilizados para mensuração da qualidade cirúrgica no contexto brasileiro. Foram selecionados indicadores de agências reguladoras do setor de saúde^10,11^; Indicadores de Segurança do Paciente (Projeto ISEP-Brasil)^12^; Projeto de Avaliação do Desempenho do Sistema de Saúde (PROADESS)^13^ e do Centro Colaborador para Qualidade e Segurança do Paciente (PROQUALIS)^14^.

**Etapa 2 - Seleção dos indicadores para consenso:** A partir dos indicadores encontrados na etapa anterior, foram selecionados aqueles que possuíam os seguintes critérios: a) aspectos relacionados a todo o processo cirúrgico; b) evidência científica alta (1A); c) servem para avaliar a qualidade da assistência cirúrgica em qualquer hospital do sistema nacional de saúde; d) podem ser usados para implementar medidas de melhoria com base em seus resultados. Excluíram-se indicadores similares, sentinelas, não aplicados ao contexto do SUS, que avaliam procedimento cirúrgico ou grupo de pacientes específico, com evidências contraditórias e indicadores que apresentam dificuldades de medição (muitos componentes de medidas, pouco claro).

**Etapa 3 - Validação de conteúdo dos indicadores:** Foi realizada mediante uso do método RAND/UCLA^15^, metodologia que associa aspectos dos métodos Delphi e de Grupo Nominal^14^, e combina a observação das evidências científicas disponíveis com o julgamento coletivo de especialistas. A validação dos indicadores é feita por meio de uma opinião de consenso derivada de um grupo, com opiniões individuais agregadas, sendo esta uma abordagem estabelecida para o desenvolvimento de indicadores de saúde^5^. O grupo de especialistas foi composto por oito cirurgiões e duas enfermeiras. Nove integrantes desse grupo de especialistas trabalhavam em instituições públicas em quatro estados brasileiros distintos, e um era um cirurgião espanhol que coordenou estudo semelhante em seu país.

Foram estabelecidas duas rodadas de consenso: a primeira ocorreu por meio do preenchimento do questionário eletrônico enviado por e-mail e segunda ocorreu por webconferência.

Foi desenvolvido um questionário utilizando a plataforma *Google Forms,* baseado em estudos semelhantes^12,14,16^, contendo cinco perguntas fechadas para cada indicador, utilizando a escala Likert para resposta. Foram utilizados, para a avaliação e seleção dos indicadores, os seguintes critérios: 1) o indicador é claramente relevante?; 2) o indicador mede a qualidade do cuidado ou segurança na assistência cirúrgica?; 3) o indicador pode ser modificado com intervenções de melhoria implementadas pelo hospital?; 4) os dados para a mensuração do indicador são possíveis de coletar?; e 5) a redação do indicador é clara, com terminologia correta e não deixa dúvidas?

Os indicadores que obtivessem um índice de validação de conteúdo (IVC) maior que 80%^17^ nos cinco itens propostos seriam considerados válidos para a mensuração da qualidade cirúrgica. Os indicadores que não atingissem esse valor na primeira rodada seriam levados para a segunda rodada.

Como subsídio para as duas rodadas, foi desenvolvida a ficha dos indicadores, contendo as informações: título, medida, justificativa, tipo do indicador, fonte de dados, descrição do numerador e denominador, esclarecimentos/definição dos termos, limitações/exceções e referências bibliográficas.

**Etapa 4 - Estudo piloto para análise da confiabilidade:** Para análise de confiabilidade dos indicadores de processo, foi realizado estudo piloto em um hospital da rede estadual de saúde do RN. Três amostras foram estabelecidas a partir do conjunto de cirurgias descrito no Sistema de Gerenciamento da Tabela de Procedimentos (SIGTAP) do SUS. **Amostra 1 (A1):** Todos os procedimentos do grupo procedimentos cirúrgicos, exceto os subgrupos pequenas cirurgias e cirurgias de pele, tecido subcutâneo e mucosa, cirurgia das vias aéreas superiores, cirurgia do aparelho da visão, cirurgia obstétrica e outras cirurgias; **amostra 2 (A2):** Procedimentos cirúrgicos do subgrupo cirurgias do aparelho digestivo (cirurgias de cólon e reto); **amostra 3 (A3):** Procedimentos cirúrgicos do subgrupo cirurgias do aparelho osteomuscular (artroscopia e prótese de joelho).

A coleta foi realizada por dois avaliadores independentes, com experiência anterior em coleta de dados em prontuários, de forma transversal, em amostras de 30 prontuários cada, referentes a cirurgias eletivas ocorridas em 2020, selecionadas aleatoriamente de forma sistemática^18,19^. A adequação dos indicadores por tipo de amostra foi estabelecida mediante consulta a especialistas. A maioria dos indicadores de processo foi avaliada na amostra A1, com exceção dos indicadores “Retirada oportuna das sondas nasogástricas operatórias” e “Remoção precoce do cateter vesical”, que foram avaliados na amostra 2.

Para a análise da confiabilidade interobservador, foi calculado o índice de Kappa, para identificar o nível de concordância segundo os parâmetros estabelecidos por Landis e Koch^20^: concordância pobre (Kappa < 0.00), concordância leve (0,00 ≤ Kappa ≤ 0,20), concordância justa (0,21 ≤ Kappa ≤ 0,40), concordância moderada (0,41 ≤ Kappa ≤ 0,60), concordância substancial (0,61 ≤ Kappa ≤ 0,80) e concordância perfeita (0,81 ≤ 1.00).

**Etapa 5 - Identificação de mecanismo de tabulação para os indicadores de resultado para que possam ser monitorados via sistemas de informações oficiais** - Os indicadores de resultados validados foram analisados quanto à sua possibilidade de monitoramento por meio da utilização dos dados dos sistemas de informações oficiais, a partir da identificação de mecanismo de tabulação para o TabWin/DataSus com o banco de dados do Sistema de Informações Hospitalares do SUS (SIH-SUS).

A pesquisa foi executada sob aprovação do Comitê de Ética em Pesquisa da Universidade Federal do Rio Grande do Norte (CEP-HUOL, CAAE: 39976920.6.0000.5292), seguindo os preceitos éticos em pesquisa com seres humanos, de acordo com a resolução CNS/MS 466/12.

## RESULTADOS

Foram encontrados 217 indicadores de qualidade ou segurança relacionados a procedimentos cirúrgicos, totalizando 183 indicadores de processo e 34 indicadores de resultado. A opção por utilizar o conteúdo das revisões sistemáticas como referência principal para a busca bibliográfica se deu na perspectiva de evitar a repetição de um estudo recente com objetivos parecidos.

Dos 183 indicadores de processo, 138 foram excluídos pelo critério da baixa evidência científica (< 1A) (Figura 1). Embora o nível de evidência do indicador “Utilização de lista de verificação de cirurgia segura” não seja elevado, os pesquisadores resolveram manter esse indicador no estudo devido à sua regulamentação nos serviços de saúde brasileiros. 12 indicadores foram excluídos por serem considerados similares, dois por não serem aplicados ao SUS, cinco por serem indicadores aplicados a um público ou procedimento muito específico, oito não permitiam o desenvolvimento de ciclos de melhoria e dois eram baseados em evidências científicas contraditórias.

**Figura 1:** Fluxo seleção de indicadores segundo critérios de exclusão.

Quanto aos indicadores de resultado, 10 indicadores foram excluídos por serem considerados similares, oito eram muito específicos, dois não possibilitaram o desenvolvimento de ciclos de melhoria e quatro estavam relacionados a evento sentinela. Ao final desse processo, 16 indicadores de processo e 10 indicadores de resultado foram submetidos à validação de conteúdo junto ao grupo de especialistas. O fluxo de seleção dos indicadores pode ser observado na figura 1.

Na primeira rodada, que teve participação de 100% dos especialistas convidados, foram enviados, por e-mail, os questionários de validação, e 26 indicadores foram apresentados ao grupo. Nessa rodada, os 13 indicadores que receberam IVC maior que 80% foram considerados válidos para mensuração da qualidade cirúrgica no âmbito do SUS. Os outros 13 indicadores, por alcançarem IVC igual ou inferior a 80% em qualquer um dos critérios avaliados, foram submetidos a segunda rodada de consenso. Essa etapa ocorreu mediante webconferência e teve adesão de 80% dos especialistas convidados. Na ocasião, ocorreram discussões sobre os indicadores com IVC ≤ 80% e, posteriormente, realizada nova avaliação, conforme pode ser observado no Quadro 1.

**Quadro 1:** Resultado do índice de validade de conteúdo obtido nas fases de consenso.

Indicador	Índice de validação de conteúdo									
	I. O indicador é claramente relevante?		II.O indicador mede a qualidade do cuidado ou segurança na assistência cirúrgica?		III. O indicador pode ser modificado com intervenções de melhoria implementadas pelo hospital?		IV. Os dados para a mensuração do indicador são passíveis de coleta?		V. A redação do indicador é clara, com terminologia correta e não deixa dúvidas? Se não, por favor, sugira alteração	
Indicadores de processo										
	1ª (%)	2ª (%)	1ª (%)	2ª (%)	1ª (%)	2ª (%)	1ª (%)	2ª (%)	1ª (%)	2ª (%)
Uso de medicação analgésica ou sedação pré-operatória mediante triagem adequada	90	100	90	100	100	100	80	87,50	70	87,50
Utilização adequada de morfina perioperatória	70	87,50	50	87,50	70	100	60	87,50	70	87,50
Triagem de delirium pós-operatório	90	87,50	80	87,50	100	87,50	70	87,50	80	87,50
Controle da normotermia no perioperatório	100		100		100		90		100	
Antibioticoprofilaxia adequada	100		100		100		100		100	
Tricotomia com aparador ou tesoura	100	100	90	100	100	100	80	100	100	100
Profilaxia de tromboembolismo venoso perioperatório adequada	100		100		100		100		100	
Deambulação precoce no pós-cirúrgico	100	100	100	100	100	100	80	87,50	90	100
Alta pós-operatória com avaliação pós-operatória, profilaxia de tromboembolismo venoso e reabilitação pós-cirúrgica	100	100	100	100	100	100	80	100	90	100
Uso de carboidratos orais no pré-operatório	80	87,50	90	100	90	87,50	80	75	80	75
Recuperação aprimorada	90	87,50	90	87,50	90	87,50	70	75	80	75
Liberação da dieta oral ou por sonda enteral nas primeiras 24h	100	100	90	100	90	87,50	90		80	87,50
Retirada oportuna das sondas nasogástricas operatórias	100		100		100		100		100	
Remoção precoce do cateter vesical	100		100		100		100		100	
Registro de pressão e tempo durante isquemia controlada em cirurgia	90		90		90		90		90	
Utilização de lista de verificação de cirurgia segura	100		100		90		100		100	
Indicadores de resultado										
	1ª (%)	2ª (%)	1ª (%)	2ª (%)	1ª (%)	2ª (%)	1ª (%)	2ª (%)	1ª (%)	2ª (%)
Complicações relacionadas à anestesia	100	87,50	100	100	80	87,50	90		90	87,50
Embolia pulmonar ou trombose venosa profunda perioperatório	100		100		90		90		100	
Sepse pós-operatória	100		100		90		100		100	
Infecção do sítio cirúrgico	100		100		100		100		100	
Acidente vascular cerebral no pós-cirúrgico	80	87,50	80	87,50	70	75	80		90	87.5
Mortalidade cirúrgica em até 30 dias	100		100		90		90		90	
Admissão não programada em unidade de terapia intensiva	80	87,50	80	87,50	80	75	80		80	87,50
Readmissão hospitalar em até 30 dias	100	100	100	100	80	87	100		90	100
Tempo de internação sem mortalidade intra-hospitalar	90		90		90		100		100	
Tempo de internação com mortalidade intra-hospitalar	90		90		90		100		100	

Ao término da segunda rodada, quatro indicadores receberam IVC ≤ 80% e não foram considerados válidos: o indicador “Uso de carboidratos orais no pré-operatório”, que apresentou IVC de 75% no critério relacionado à redação do indicador; o indicador “Recuperação aprimorada” teve IVC de 75% nos critérios relacionados à disponibilização dos dados para mensuração e clareza na redação; os indicadores “Acidente vascular cerebral no pós-cirúrgico” e “Admissão não programada em unidade de terapia intensiva” obtiveram IVC de 75% nos critérios relacionados à disponibilização de dados e à possibilidade de modificação do indicador mediante intervenções de melhoria. Desse modo, 22 indicadores foram considerados válidos para a mensuração da qualidade em cirurgias, sendo 14 de processo e 8 de resultado. A fonte dos dados, o numerador e o denominador desses indicadores estão descritos no Quadro 2.

**Quadro 2:** Indicadores validados por especialistas com descrição de seus respectivos numerador, denoninador e fonte de dados.

Indicarores	Numerador	Denominador	Fonte de dados
1. Uso de medicação analgésica ou sedação pré-operatória mediante triagem adequada	Nº de pacientes que utilizaram analgésicos opióides ou sedação pré-operatória para manejo da dor e da ansiedade pré-operatória mediante triagem adequada x 100	Nº de pacientes submetidos ao uso de ansiolíticos ou analgésicos opióides no pré-operatório	Prontuário do paciente
2. Utilização adequada de morfina perioperatória	Nº de pacientes que fizeram uso de morfina ou derivados por via peridural ou raquimedular intraoperatória x 100	Nº de pacientes que fizeram uso de morfina ou derivados intraoperatórios	Prontuário do paciente
4. Controle da normotermia no intraoperatório	Nº de adultos submetidos à cirurgia com anestesia geral ou regional que tiveram normotermia (temperatura > 36°) mantida antes, durante e após a cirurgia x 100	Nº de adultos submetidos a cirurgia com anestesia geral ou regional	Prontuário do paciente
5. Antibioticoprofilaxia adequada	Nº de pacientes com profilaxia adequada em todos os seus aspectos x 100	Nº de pacientes cirúrgicos com indicação de profilaxia antibiótica cirúrgica	Prontuário do paciente
6. Tricotomia com aparador ou tesoura	Nº de cirurgias que realizaram tricotomia com aparador ou tesoura x 100	Nº de cirurgias com realização de tricotomia	Prontuário do paciente
7. Profilaxia de tromboembolismo venoso perioperatório adequada	Nº de pacientes submetidos a cirurgias com indicação tromboprofilaxia farmacológica que receberam profilaxia tromboembolismo venosa apropriada iniciada dentro de 24h antes da cirurgia até 24h após a cirurgia x 100	Nº de pacientes cirúrgicos com indicação tromboprofilaxia farmacológica	Prontuário do paciente
8. Deambulação precoce no pós-cirúrgico	Nº de pacientes que receberam orientações para deambulação precoce pós-cirúrgica x 100	Nº de pacientes cirúrgicos	Prontuário do paciente
9. Alta pós-operatória com avaliação pós-operatória, profilaxia de tromboembolismo venoso e reabilitação pós-cirúrgica.	Nº de pacientes que receberam avaliação pós-operatória, profilaxia de tromboembolismo venoso e reabilitação pós-cirúrgica antes da alta e que possuem conformidade adequada com os 3 itens x 100	Nº de pacientes com pós-operatório cirúrgico e que receberam alta	Prontuário do paciente
10. Liberação da dieta oral ou por sonda enteral nas primeiras 24h	Nº de pacientes que tiveram a dieta liberada por via oral ou SNE nas primeiras 24h x 100	Nº de pacientes submetidos a procedimentos cirúrgicos	Prontuário do paciente
11. Retirada oportuna das sondas nasogástricas operatórias	Nº de pacientes os quais retiraram a sonda nasogástrica antes do término da cirurgia	Nº de pacientes submetidos a procedimentos cirúrgicos gastrointestinais e que demandaram uso de sonda nasogástrica para drenagem	Prontuário do paciente
12. Remoção precoce do cateter vesical	Nº de pacientes cujo cateter foi retirado até 48 horas do pós-operatório x 100	Nº de pacientes cirúrgicos que utilizaram cateter vesical	Prontuário do paciente
13. Registro de pressão e tempo durante isquemia controlada em cirurgia	Nº de intervenções com registro do tempo e pressão adequados do torniquete pneumático X 100	Nº de intervenções cirúrgicas com torniquete pneumático	Prontuário do paciente
14. Utilização de lista de verificação de cirurgia segura	N° de cirurgias com preenchimento completo do checklist de segurança da OMS x 100	Nº de cirurgias realizadas na instituição	Prontuário do paciente
15. Complicações relacionadas à anestesia	Altas com efeitos adversos anestésicos, ou intoxicação anestésica, registrados nos diagnósticos secundários, dentre os casos que preencham os critérios de inclusão e exclusão do denominador	Todas as altas cirúrgicas, de pacientes com 18 anos ou mais	SIH-SUS
16. Embolia pulmonar ou trombose venosa profunda perioperatória	Altas, no diagnóstico secundário, de trombose venosa profunda ou embolia pulmonar, entre os casos que preencham os critérios de inclusão e exclusão do denominador x 100	Todas as altas cirúrgicas de pacientes com 18 anos ou mais	SIH-SUS
17. Sepse no pós-operatória	Altas de sepse no diagnóstico secundário, entre os casos que preencham os critérios de inclusão e exclusão do denominador x 1000	Todas as altas cirúrgicas de pacientes com 18 anos ou mais	SIH-SUS
18. Infecção do sítio cirúrgico	Nº de infecções de sítio cirúrgico (em até 30 dias) x 100	Nº de cirurgias realizadas no período	Prontuário do paciente
19. Mortalidade cirúrgica em até 30 dias	Nº de óbitos cirúrgicos observados no hospital	Nº de procedimentos cirúrgicos realizados no hospital	SIH-SUS
20. Readmissão hospitalar por complicações pós-operatórias relacionadas ao procedimento cirúrgico	Nº de pacientes readmitidos entre 0 e 29 dias da alta hospitalar pós-procedimento cirúrgico com complicações relacionadas à cirurgia x 100	Nº de altas cirúrgicas	SIH-SUS
21. Tempo de internação sem mortalidade intra-hospitalar	Somatório do nº de dias que cada paciente que recebe alta sem óbito passa internado após um procedimento cirúrgico	Somatório do nº de pacientes que ficaram internados após um procedimento operatório e não evoluem a óbito	SIH-SUS
22. Tempo de internação com mortalidade intra-hospitalar	Somatório do nº de dias que cada paciente passou internado	Somatório do nº de pacientes que ficaram internados após um procedimento operatório e evoluem a óbito	SIH-SUS

SIH-SUS: Sistema de Informações Hospitalares do SUS.

As fichas de qualificação dos indicadores validados foram reformuladas de acordo com as sugestões dos especialistas, com a adição e reformulação de termos e conceitos.

Para análise da confiabilidade dos indicadores, cuja fonte de dados são os prontuários, realizou-se um estudo piloto retrospectivo no Hospital Regional Mariano Coelho (HRMC), em Currais Novos/RN, nos meses de setembro e outubro de 2021. O HRMC é um hospital que possui 32 leitos cirúrgicos habilitados, referência na realização procedimentos cirúrgicos eletivos para a região de saúde em que está inserido.

Devido ao perfil de habilitação do HRMC, não foi possível realizar a coleta dos indicadores “Alta pós-operatória com avaliação pós-operatória, profilaxia de tromboembolismo venoso e reabilitação pós-cirúrgica”, e “Registro de pressão e tempo durante isquemia controlada em cirurgia”. Foi cogitada a busca de outra instituição da rede estadual hospitalar que fosse habilitada para realização de cirurgias ortopédicas para avaliação desses indicadores; entretanto, não foi possível, diante do baixo número de cirurgia eletivas ortopédicas realizadas no ano de 2020, em virtude da pandemia de covid-19, além da inexistência de torniquete pneumático nas instituições hospitalares que compõem a rede estadual.

Quanto à análise de confiabilidade, seis indicadores apresentaram confiabilidade substancial e dois confiabilidade quase perfeita^20^, como pode ser observado na Tabela 1. Não foi possível medir a confiabilidade para os indicadores de processo “Controle de normotermia no perioperatório”, “Triagem de delirium pós-operatório”, “Profilaxia de tromboembolismo venoso perioperatório adequada” e “Utilização de lista de verificação de cirurgia segura”, uma vez que o percentual de conformidade para esses indicadores foi de 0% para os dois avaliadores.

**Tabela 1:** Análise da confiabilidade dos indicadores de qualidade cirúrgica segundo os parâmetros de Landis e Koch (1977) e percentual de conformidade alcançado.

Variável	Índice de Kappa^a^	Classificação^b^	Prevalênica da característica avaliada
Não-administração rotineira de medicação anestésica ou sedação pré-operatória	0,73	Substancial	46,7
Utilização adequada de morfina perioperatória	0,66	Substancial	53,3
Triagem de delirium pós-operatório	-	-	0
Controle da normotermia no perioperatório	-	-	0
Antibioticoprofilaxia adequada	0,62	Substancial	23,3
Tricotomia com aparador ou tesoura	1	Quase perfeita	100
Profilaxia de tromboembolismo venoso perioperatório adequada	-	-	0
Deambulação precoce no pós- cirúrgico	0,72	Substancial	33
Alta pós-operatória com avaliação pós-operatória, profilaxia de tromboembolismo venoso e reabilitação pós-cirúrgica	-	-	-
Liberação da dieta oral ou por sonda enteral nas primeiras 24h	0,76	Substancial	80
Utilização adequada das sondas nasogástricas (SNG) pós-operatórias	0,65	Substancial	96,7
Remoção precoce do cateter vesical	1	Quase perfeita	100
Registro de pressão e tempo durante isquemia controlada em cirurgia	-	-	-
Utilização de lista de verificação de cirurgia segura	-	-	0
Infecção de sítio cirúrgico	-	-	Obs.: não foram observados registros de eventos nos prontuários investigados

^a^ Para todos os casos p < 0,001

^b^ Grau de concordância inter-avaliador.

Para os indicadores de resultado, cuja fonte de dados é o SIH-SUS, foi observado que sete dos oito indicadores validados podem ser monitorados a partir do tabulador TabWin/DATASUS). Os dados são de acesso público e estão disponíveis no endereço eletrônico https://datasus.saude.gov.br/transferencia-de-arquivos/.

Não foi possível realizar tabulação para o indicador “Readmissão pós-cirúrgica”. Por se tratar de um sistema que analisa a produção hospitalar, ele não vincula as internações a um registro individual do usuário, ou seja, por meio do sistema não é possível identificar quantas vezes um único usuário foi admitido no hospital, tampouco é possível averiguar se uma admissão estaria relacionada à outra anterior.

Foi elaborado instrutivo de tabulação dos indicadores de resultado para o aplicativo TabWin/DATASUS para as equipes que farão a coleta de dados e monitoramento. Todos os resultados obtidos com os outros indicadores podem ser observados na Tabela 2.

**Tabela 2:** Estimativas dos indicadores de resultado.

Indicador	Eventos	Denominador	Resultado
Complicações relacionadas à anestesia	0	631 procedimentos cirúrgicos realizados	0
Embolia pulmonar ou trombose venosa profunda perioperatório	0	631 procedimentos cirúrgicos realizados	0
Sepse pós-operatória	0	631 procedimentos cirúrgicos realizados	0
Infecção do sítio cirúrgico	0	631 procedimentos cirúrgicos realizados	0
Mortalidade cirúrgica em até 30 dias	4 óbitos pós-cirúrgicos	631 procedimentos cirúrgicos realizados	0,0063
Readmissão hospitalar	-	631	0
Tempo de internação com mortalidade intra-hospitalar	48 diárias com óbitos pós-cirúrgicos	4 altas com óbitos após procedimento cirúrgico eletivo	12 dias de permanência em média
Tempo de internação sem mortalidade intra-hospitalar	974 diárias sem óbitos pós-cirúrgicos	627 altas sem óbito após de procedimentos cirúrgicos eletivos	1,5 dias de permanência em média

## DISCUSSÃO

O estudo contribuiu para o desenvolvimento de um conjunto de 22 indicadores com alto nível de evidência, que passaram por processo rigoroso de validação de conteúdo para possibilitar o monitoramento da qualidade da assistência cirúrgica no âmbito do SUS. Esses indicadores poderão orientar a gestão da instituição e a gestão da rede hospitalar como um todo, identificando as fragilidades que devem ser trabalhadas, visando a oferta de uma assistência segura à população. Trata-se, então, de conjunto inicial de indicadores altamente relevantes para o monitoramento e a melhoria da qualidade da assistência cirúrgica no âmbito do SUS do RN com possibilidade de ser utilizado por qualquer outro serviço de saúde.

A partir dos indicadores de processo, é possível avaliar todas as etapas e atividades realizadas na implementação de um tratamento ou episódio de cuidado^8^. Logo, monitorar continuamente esses indicadores possibilita identificar fragilidades na prestação do cuidado. Segundo Donabedian, os indicadores de processo são a única medida direta da qualidade, pois a estrutura pode não ser utilizada e os resultados podem se dever a outros fatores para além da boa assistência^21^.

O monitoramento dos indicadores de resultado “Mortalidade pós- cirúrgica”, “Readmissão pós-cirúrgica” e “Tempo médio de internação com e sem óbito”, por meio do sistema de informação, possibilita a mensuração da qualidade de um serviço de saúde isolado, como também viabiliza o *benchmarking*. Ou seja, possibilita a comparação de serviços de saúde da rede hospitalar estadual e também em nível nacional, o que fortalece os sistemas de informação^22^.

O indicador de mortalidade pós-cirúgica está entre os indicadores propostos pela Comissão Lancet^23^ para avaliar os cuidados cirúrgicos. Estudo similar^16^ desenvolvido para o sistema de saúde espanhol apontou também os indicadores: “Readmissão pós-cirúrgica”, “Profilaxia de tromboembolismo venoso”, “Antibioticoprofilaxia adequada” e “Infecção do sítio cirúrgico” como indicadores válidos para avaliar a qualidade cirúrgica; entretanto, estes indicadores são direcionados apenas para cirurgias do aparelho digestivo.

O *benchmarking* vem sendo utilizado para buscar oportunidades de melhoria e fazer comparações de organizações semelhantes^16,24^. Foi listado como estratégia pela Organização Mundial da Saúde (OMS) no Plano de ação global para a segurança do paciente 2021-2030^22^, e o desenvolvimento de “bons” indicadores é um fator de sucesso para ações de *benchmarking*^25^.

Outrossim, 11 indicadores puderam ser medidos com as fontes de dados disponíveis (prontuários e dados do sistema de informação oficial), sendo os 8 indicadores de processo avaliados em prontuários e 3 indicadores de resultado medidos com dados do SIH-SUS, explorando a viabilidade do emprego desse sistema para avaliar a qualidade da assistência cirúrgica. Para os indicadores “Triagem de delirium pós-operatório”, “Utilização de lista de verificação de cirurgia segura” e “Profilaxia de tromboembolismo venoso perioperatória adequada”, faz-se necessária a institucionalização de protocolos relacionados a esses indicadores, o que sinaliza uma oportunidade de melhoria para o hospital onde foi desenvolvido o piloto.

A confiabilidade interavaliadores, testada pela estatística Kappa para oito indicadores de processo, encontrou valores que caracterizam um grau de confiabilidade substancial e quase perfeita, o que reforça a solidez desses indicadores. O teste de Kappa é considerado adequado para avaliar a confiabilidade de interavaliadores de variáveis categóricas e nominais, e é utilizado com frequência para avaliar a confiabilidade neste tipo de estudo^20^.

Para o indicador Infecção do Sítio Cirurgico (ISC), cujas fontes de dados podem ser prontuários ou dados do sistema, não foi possível analisar a confiabilidade, uma vez que não foi observado o evento nos prontuários selecionados para compor a amostra. A maioria das ISC, ocorre, em média, de quatro a seis dias após o procedimento, e a média do tempo de internação para os procedimentos incluídos no estudo foi de 1,5 dias de permanência. Estudos apontam que, nos procedimentos em que o tempo de permanência pós-operatório é curto, os dados de ISC, obtidos somente de pacientes internados, não refletem a real ocorrência de infecção^26^. Observou-se um aumento da ISC de quatro vezes quando a vigilância pós-alta foi realizada^27^, o que nos leva à constatação de que o prontuário do paciente não se revela como a melhor fonte de dados para o monitoramento desse indicador para a grande maioria dos procedimentos realizados pelo SUS.

Para os indicadores de resultado “Complicações relacionadas à anestesia”, “Sepse pós-operatória”, “Edema pulmonar ou trombose venosa profunda”, não foi possível a mensuração via sistema de informação. Os resultados foram nulos, possivelmente em virtude de sub-registro de eventos secundários nas fichas de Autorização de Internação Hospitalar (AIH). Um estudo sobre a confiabilidade dos dados da AIH no país identificou elevado grau de sub-registro de diagnóstico secundário na AIH^28^. O sub-registro de diagnóstico secundário nas internações cirúrgicas impacta na precisão das medidas calculadas para esses indicadores, o que se configura como uma oportunidade de melhoria para o sistema de informações em saúde.

O Conjunto Mínimo de Dados da Atenção à Saúde (CMD), idealizado em 2015, é uma estratégia assumida pelos gestores das três esferas de gestão do SUS para redução da fragmentação dos sistemas de informação, e substituiria os principais sistemas de informação da atenção à saúde do país. Entretanto, apesar de ter sido oficialmente instituído por resolução da Comissão Intergestores Tripartite^29^, ainda não teve sua implantação concluída. A implantação do CMD possibilitaria a utilização de dados administrativos, clínico-administrativos e clínicos por meio de um único documento, além de possibilitar análises mais específicas, uma vez que relacionaria as informações à identificação dos usuários por meio da integração com a base do sistema Cartão Nacional de Saúde. Apesar dos esforços e trabalhos desenvolvidos na área da segurança do paciente, a capacidade para reduzir o risco, evitar danos e melhorar a segurança dos cuidados de saúde ainda é prejudicada pela ausência de sistemas de informação de alta qualidade^22^.

A revisão da literatura existente e os métodos de consenso são cada vez mais utilizados e recomendados pela comunidade científica para este tipo de estudo^16,30^. A utilização de método RAND/UCLA para o estabelecimento de consenso, mediante o uso de recursos de comunicação à distância (*internet*), permitiu reunir especialistas qualificados de várias regiões do país. O interesse dos especialistas na área estudada, associado aos índices de consenso observados, conferiu credibilidade aos resultados, como pode ser observado em outros estudos^14,31^.

Como limitações deste estudo, podemos destacar a realização do estudo piloto em um único hospital, cujo perfil de assistência não incluiu procedimentos cirúrgicos do aparelho osteomuscular e a realização do estudo piloto em um período pandêmico, o que diminuiu o universo amostral, em virtude do cancelamento de cirurgias eletivas em toda a rede hospitalar. Outras limitações, que podem ser alvos de novos estudos, são a não avaliação de indicadores de estrutura e a não realização da análise da factibilidade para coleta dos indicadores.

## CONCLUSÃO

O estudo contribuiu com o desenvolvimento de um conjunto de indicadores de qualidade no âmbito cirúrgico, que se traduz como mecanismo eficaz de mensuração do desempenho e da qualidade dos serviços ofertados pela rede de serviços hospitalares do RN e do Brasil. São 22 indicadores que foram considerados válidos, sendo 8 indicadores de processo considerados confiáveis e sete indicadores de resultados, em que foram identificados parâmetros para tabulação utilizando os sistemas de informações oficiais. Esse conjunto de indicadores possibilita a documentação da qualidade do cuidado, viabiliza comparações e o *benchmarking* entre unidades de saúde, promove a identificação de prioridades, por meio do fortalececimento e otimização das estratégias de monitoramento e de melhorias dirigidas à segurança do paciente em hospitais do SUS.

Logo, trata-se de uma proposta inovadora, compatível com a realidade brasileira, para orientar gestores públicos e pesquisadores no processo de monitoramento da qualidade cirúrgica.
